# A phase I dose-escalation study of TAK-733, an investigational oral MEK inhibitor, in patients with advanced solid tumors

**DOI:** 10.1007/s10637-016-0391-2

**Published:** 2016-09-21

**Authors:** Alex A. Adjei, Patricia LoRusso, Antoni Ribas, Jeffrey A. Sosman, Anna Pavlick, Grace K. Dy, Xiaofei Zhou, Esha Gangolli, Michelle Kneissl, Stephanie Faucette, Rachel Neuwirth, Viviana Bózon

**Affiliations:** 1 Department of Oncology, Mayo Clinic, 200 First St, SW, Rochester, MN 55905 USA; 2 Yale University, New Haven, CT USA; 3 University of California at Los Angeles Jonsson Comprehensive Cancer Center, Los Angeles, CA USA; 4 Vanderbilt-Ingram Cancer Center, Nashville, TN USA; 5 New York University Langone Medical Center, New York, NY USA; 6 Roswell Park Cancer Institute, Buffalo, NY USA; 7 Millennium Pharmaceuticals, Inc., a wholly owned subsidiary of Takeda Pharmaceutical Company Limited, Cambridge, MA USA; 8 AstraZeneca Pharmaceuticals, Waltham, MA USA; 9 Present address: Array BioPharma Inc., Boulder, CO USA

**Keywords:** TAK-733, MEK inhibition, Phase 1, Solid tumor

## Abstract

*Purpose* TAK-733, an investigational, selective, allosteric MEK1/2 inhibitor, has demonstrated antitumor effects against multiple cancer cell lines and xenograft models. This first-in-human study investigated TAK-733 in patients with solid tumors. *Methods* Patients received oral TAK-733 once daily on days 1–21 in 28-day treatment cycles. Adverse events (AEs) were graded using the Common Terminology Criteria for AEs version 3.0. Response was assessed using RECIST v1.1. Blood samples for TAK-733 pharmacokinetics and pharmacodynamics (inhibition of ERK phosphorylation) were collected during cycle 1. *Results* Fifty-one patients received TAK-733 0.2–22 mg. Primary diagnoses included uveal melanoma (24 %), colon cancer (22 %), and cutaneous melanoma (10 %). Four patients had dose-limiting toxicities of dermatitis acneiform, plus fatigue and pustular rash in one patient, and stomatitis in one patient. The maximum tolerated dose was 16 mg. Common drug-related AEs included dermatitis acneiform (51 %), diarrhea (29 %), and increased blood creatine phosphokinase (20 %); grade ≥ 3 AEs were reported in 27 (53 %) patients. Median T_max_ was 3 h; systemic exposure increased less than dose-proportionally over the dose range 0.2–22 mg. On day 21 maximum inhibition of ERK phosphorylation in peripheral blood mononuclear cells of 46–97 % was seen in patients receiving TAK-733 ≥ 8.4 mg. Among 41 response-evaluable patients, 2 (5 %) patients with cutaneous melanoma (one with BRAF L597R mutant melanoma) had partial responses. *Conclusions* TAK-733 had a generally manageable toxicity profile up to the maximum tolerated dose, and showed the anticipated pharmacodynamic effect of sustained inhibition of ERK phosphorylation. Limited antitumor activity was demonstrated. Further investigation is not currently planned.

**Electronic supplementary material:**

The online version of this article (doi:10.1007/s10637-016-0391-2) contains supplementary material, which is available to authorized users.

## Introduction

The Ras/Raf/MEK/ERK mitogen-activated cascade plays a central role in the signaling required for cell proliferation, survival, motility, differentiation, and angiogenesis [1]. Dysregulation or hyperactivation of this cascade is common in many human cancers [1, 2]. In particular, MEK is frequently activated in cancers with mutations in established upstream oncogenes [3, 4]; specifically, mutations in *RAS* and *RAF* oncogenes can lead to increased MEK activation [3]. The *RAS* gene family members include *HRAS*, *KRAS*, and *NRAS*, with the latter two being the isoforms commonly mutated in cancers. *RAS* activating mutations occur in 30 % of all cancers, including a high prevalence in melanoma (15–25 %) [3, 5], with *KRAS* mutations more common in adenocarcinomas and solid tumors and *NRAS* mutations more common in leukemia, thyroid carcinoma, and malignant melanoma [6]. *RAS*, specifically *KRAS* [6], is frequently mutated in colorectal cancer (CRC) and has been linked to CRC initiation and progression [7, 8]. Furthermore, approximately 8 % of human tumors have mutations in *BRAF* (a member of the *RAF* family) — melanoma, thyroid cancer, and CRC have been associated with a high frequency of *BRAF* mutations [9, 10]. Specifically, the V600E point mutation accounts for more than 80 % of *BRAF* activating mutations [9, 10].

Therefore, given this background, MEK is a potential therapeutic target of interest for pharmacologic intervention in cancer. Inhibition of MEK has been shown to impair cell proliferation and impact a diverse array of cellular events including differentiation, apoptosis, and angiogenesis [11–15]. A number of MEK1/2 inhibitors are currently being investigated in the clinic across a range of cancers [16–19] including gynecologic malignancies [20], melanoma [17, 21], colorectal cancer [17], and acute myelogenous leukemia [22], with trametinib approved alone and in combination with the BRAF inhibitor dabrafenib for advanced metastatic melanoma with *BRAF* V600 mutations [23].

TAK-733 is an investigational, orally available, selective, non-ATP competitive, allosteric inhibitor of MEK1/2 with an IC_50_ for MEK signaling inhibition of 2–5 nM [24]. In the preclinical setting, TAK-733 has exhibited antitumor effects in vitro and in vivo against multiple cancer cell lines and xenograft models. For example, TAK-733 has demonstrated activity against multiple cutaneous melanoma cell lines, with a high proportion of *BRAF* V600E-mutant cell lines showing high sensitivity (IC_50_ < 0.1 μM) and with no statistically significant association between BRAF status and response [25], and against uveal melanoma cell lines [26]. Additional studies have also shown tumor growth inhibition and regressions with TAK-733 (dosed once daily) in human melanoma explant mouse models and mouse xenograft models [25, 27]. Synergistic activity was seen with TAK-733 in combination with the pan-RAF inhibitor TAK-632 in both *BRAF*-mutated melanoma cells and *NRAS*-mutated melanoma cells with acquired resistance to BRAF inhibitors [28]. Furthermore, antitumor activity has been reported in mesothelioma cell lines [29], and tumor growth suppression has also been seen in patient-derived colorectal cancer human tumor explants [30], and in mouse xenograft models of colorectal cancer [27] and lung cancer [31]. Pharmacokinetic data in mouse xenograft models indicate that plasma concentrations of TAK-733 decrease rapidly 8–16 h after once-daily oral dosing [25]. Demonstrated pharmacodynamic effects of MEK inhibition with TAK-733 include decreases in phosphorylated ERK (pERK) [26], as seen in both sensitive and resistant melanoma cell lines and in tumor-bearing mice; elevated TAK-733 tumor concentrations were shown to correspond approximately with pERK reductions [25].

Based on these preclinical observations, this first-in-human, multicenter, open-label, phase I, dose-escalation study (NCT00948467) was conducted to investigate the safety, tolerability, pharmacokinetics, pharmacodynamics, and preliminary activity of TAK-733 in patients with solid tumors.

## Patients and methods

### Patients

Patients aged ≥18 years with an Eastern Cooperative Oncology Group (ECOG) performance status of 0–2; a diagnosis of a nonhematologic malignancy for which there was no standard, curative, or life-prolonging treatment available; and with radiographically or clinically evaluable tumor (measurable disease as defined by the Response Evaluation Criteria for Solid Tumors [RECIST] was not a requirement during the dose-escalation stage) were eligible. Patients with ovarian or prostate cancer with elevated tumor markers (e.g. CA125 or prostate-specific antigen [PSA]) in the absence of measurable disease were also eligible. Patients also required adequate hematologic, renal, and hepatic function that were defined as: hemoglobin ≥9 g/dL; an absolute neutrophil count (ANC) ≥1500/mm^3^; a platelet count ≥100,000/mm^3^; a prothrombin time/international normalized ratio (INR) or activated partial thromboplastin time ≤ 1.5 times the upper limit of normal (ULN); calculated creatinine clearance ≥50 mL/min; serum phosphorous or albumin-adjusted serum calcium ≤ULN; bilirubin ≤1.5 x ULN; and aspartate aminotransferase (AST), alanine aminotransferase (ALT), or alkaline phosphatase (ALP) ≤2.5 x ULN (AST, ALT, and ALP may have been elevated up to 5 x ULN if elevation could be reasonably ascribed to the presence of metastatic disease to liver and/or bone).

Patients who had major surgery within 14 days; strong or moderate CYP3A4 inhibitors/inducers within 14 days; antineoplastic therapy or radiotherapy within 21 days; any investigational product within 28 days; nitrosoureas or mitomycin C within 42 days; prior biologic or immunotherapy within 4 weeks; or prior ipilimumab within 4 months of first dose were not eligible. Patients were also excluded if they had symptomatic brain metastases; grade ≥ 2 unresolved toxicity (except alopecia) from previous anticancer treatment; an ongoing or newly diagnosed eye abnormality putting the patient at risk for retinal vein thrombosis or central serous retinopathy; cardiovascular abnormalities including abnormal left ventricular ejection fraction, electrocardiogram, uncontrolled cardiovascular condition; or if they were receiving therapeutic anticoagulation.

Institutional review boards at each of the participating investigational centers approved the study, which was conducted in accordance with the ethical principles originating in or derived from the Declaration of Helsinki and its amendments and in accordance with 21 Code of Federal Regulations 50 / 56 / 312. All patients provided written informed consent.

### Study design

This was an open-label, multicenter, first-in-human, phase I dose-escalation study. Patients were enrolled and treated at five sites in the United States from December 22, 2009, to April 30, 2013. The primary objectives were to evaluate the safety profile and determine the dose-limiting toxicities (DLTs), maximum tolerated dose (MTD), and recommended phase II dose (RP2D) of TAK-733, and to characterize the pharmacokinetics of TAK-733. The secondary objective was to evaluate antitumor activity. Exploratory objectives included investigating potential pharmacodynamic effects of TAK-733 in peripheral blood mononuclear cells, namely levels of pERK, and exploring pharmacokinetic/pharmacodynamic and pharmacokinetic/safety relationships.

Patients received oral TAK-733 once daily on days 1–21 of 28-day treatment cycles. Dose escalation initially proceeded from a starting dose of 0.2 mg in 100 % increments using a single-patient cohort design (while allowing enrollment of additional eligible patients, up to four), until any patient experienced a DLT or a drug-related grade ≥ 3 non-DLT adverse event (AE; except creatine kinase elevation) in cycle 1; or any patient experienced a TAK-733-related grade ≥ 2 AE in cycle 1. Dose escalation then proceeded in ≤40 % increments using a modified 3 + 3 cohort design. No intra-patient dose escalation was permitted. The MTD was defined as the highest dose at which cycle 1 DLTs occurred in 0/3 or 1/6 patients. If fewer than 6 patients had been enrolled, the MTD dose level was expanded to a total of 6 patients.

DLTs were defined as any of the following considered possibly related to TAK-733: grade 4 neutropenia lasting ≥7 consecutive days or grade ≥ 3 neutropenia with fever/infection; grade 4 thrombocytopenia lasting ≥7 consecutive days, platelet count <10,000/mm^3^ at any time, or grade ≥ 3 thrombocytopenia with clinically significant bleeding; grade 4 anemia; grade ≥ 3 nausea/emesis despite optimal prophylaxis or grade ≥ 3 diarrhea despite optimal antidiarrheal therapy; any other grade ≥ 3 nonhematologic toxicity (except grade ≥ 3 creatine kinase elevation considered not clinically significant); delay of >2 weeks in initiation of next cycle due to lack of adequate recovery of TAK-733-related toxicities; the inability to receive ≥75 % of planned doses in a cycle due to TAK-733-related toxicity; and any other grade ≥ 2 TAK-733-related toxicities requiring dose reduction or discontinuation.

Following the initial dose-escalation stage, an expansion stage was planned at the MTD to investigate the safety, pharmacokinetics, pharmacodynamics, and antitumor activity of TAK-733 in patients with advanced unresectable melanoma. However, due to changes in the standard of care for melanoma treatment during the conduct of this study [32], coupled with a review of pharmacologic findings and the initial clinical findings on TAK-733 from the present study, a decision was made to cancel this stage of the study following thorough consideration by the sponsor.

### Assessments

AEs were monitored throughout the trial and for 30 days after the last dose of study medication and were graded using the National Cancer Institute’s Common Terminology Criteria for AEs version 3.0. Regular ophthalmologic examination was included in the safety monitoring. Response was assessed using the modified RECIST guideline (v1.1) [33]. Blood samples for determination of TAK-733 plasma concentrations were obtained at the following time points: pre-dose, and at 0.25, 0.5, 1, 2, 3, 4, 6, 8, 10, and 24 h post-dose on days 1 and 21 of cycle 1; pre-dose on days 8 and 15 of cycle 1; 48, 72, 96, and 120 h post-dose on day 21 of cycle 1; and pre-dose on day 1 of cycle 2. Urine samples were collected over 0–24 h post-dose on day 21 of cycle 1.

Plasma and urine samples were analyzed for TAK-733 concentrations using validated liquid chromatography–tandem mass spectrometry (LC–MS/MS) methods. The methods were applicable to the quantitation of TAK-733 within a dynamic range of 0.1–200 ng/mL for plasma samples and 5–10,000 ng/mL for urine samples. Quality controls, analyzed in duplicate over the validation range, and inter-assay precision, evaluated at each level in duplicate in six runs, met the performance and acceptance criteria. Noncompartmental analyses, using WinNonlin version 6.1 or higher (Pharsight, Cary, NC), in pharmacokinetic-evaluable patients were used to determine plasma and urine pharmacokinetic parameters including: C_max_, maximum plasma concentration; T_max_, time to C_max_; AUC_0–τ_, area under the plasma concentration–time curve over the dosing interval; CL/F, apparent clearance; t_½_, terminal elimination half-life; accumulation ratio; peak/trough ratio; and renal clearance.

Blood samples for pharmacodynamic analysis (pERK levels) were collected at the following time points: pre-dose and at 0.5, 1, 2, 4, 8, and 24 h post-dose on days 1 and 21 of cycle 1; pre-dose on days 8 and 15 of cycle 1; 48, 72, 96, and 120 h post-dose on day 21 of cycle 1; and pre-dose on day 1 of cycle 2. The extent of ERK phosphorylation in peripheral blood mononuclear cells was determined using an ex vivo stimulation assay; pERK levels in CD3-positive lymphocytes were measured by flow cytometry assessing mean fluorescence (MEFL) under phorbol myristate acetate (PMA)-stimulated and unstimulated conditions. Mean percent change from baseline in blood pERK level over time was determined following single and multiple doses of TAK-733. Noncompartmental analyses, using WinNonlin version 6.1 or higher (Pharsight, Cary, NC), in pharmacodynamic-evaluable patients were used to determine the following pharmacodynamic parameters for inhibition of ERK phosphorylation: maximum observed effect (E_max_), time to E_max_ (TE_max_), area under the effect (inhibition)–time curve over the dosing interval (AUEC_(0-τ)_), and average effect over the dosing interval (E_av_).

### Statistics

The safety population included all patients who received ≥1 dose of TAK-733. The DLT-evaluable population included all patients who either experienced a DLT during cycle 1 or received ≥80 % of scheduled cycle 1 doses without experiencing a DLT. The response-evaluable population included patients with measurable disease at baseline and ≥1 post-baseline assessment. The pharmacokinetic-evaluable population included patients in the safety population for whom there were sufficient dosing and sufficient concentration–time data to reliably estimate pharmacokinetic parameters. The pharmacodynamic-evaluable population included patients in the safety population who had sufficient dosing history and sufficient tumor and/or blood pharmacodynamic data available.

## Results

### Patients

Fifty one patients were enrolled and received ≥1 dose of TAK-733. Patients’ characteristics are summarized in Table 1. The most common primary diagnoses included uveal melanoma (24 %), colon cancer (22 %), and cutaneous melanoma (10 %). Most (90 %) patients had received prior antineoplastic therapy, with 71 % having received three or more prior therapies. Although not an exclusion criterion for the dose-escalation portion of the study, no patients were reported to have been previously treated with inhibitors of the MAPK pathway. Thirty nine (76 %) patients discontinued treatment due to progressive disease, 7 (14 %) due to AEs, 2 (4 %) each due to symptomatic deterioration and patient withdrawal, and 1 (2 %) for non-compliance.

**Table 1:** Baseline patient demographics and disease characteristics. ECOG, Eastern Cooperative Oncology Group; NSCLC, non-small cell lung cancer

Characteristic	*N* = 51
Median age, years (range)	58 (24–75)
Male, *n* (%)	26 (51)
Race, *n* (%)	
White	42 (82)
Black or African American	8 (16)
Not reported	1 (2)
ECOG performance status, *n* (%)	
0	22 (43)
1	29 (57)
Disease primary diagnosis, *n* (%)	
Melanoma uveal	12 (24)
Colon cancer	11 (22)
Melanoma of the skin	5 (10)
Other melanoma*	4 (8)
NSCLC	3 (6)
Anal cancer	2 (4)
Colorectal cancer	2 (4)
Rectal cancer	2 (4)
Other^†^	10 (20)
Prior therapy, *n* (%)	
Prior surgery or non-radiation procedure	50 (98)
Prior radiation	38 (75)
Prior antineoplastic therapy	46 (90)
1	7 (14)
2	3 (6)
≥ 3	36 (71)
Best response to last prior antineoplastic therapy, *n* (%)	
Partial response	4 (8)
Stable disease	11 (22)
Progressive disease	22 (43)
Unknown	8 (16)

*Melanoma of the scapular, ocular malignant melanoma, ocular melanoma, and melanoma: unknown, each n = 1. †Adrenal, bladder, head and neck, liver, ovarian, and skin cancer, melanoma, sarcoma, unknown high-grade malignant neoplasm, and unknown primary cancer, each *n* = 1

### Dose escalation, DLTs, and MTD determination

Fifty one patients received TAK-733 at one of eleven dose levels: 0.2 mg (*n* = 1); 0.4 mg (*n* = 1); 0.8 mg (*n* = 2); 1.6 mg (*n* = 2); 3.2 mg (*n* = 4); 4.4 mg (*n* = 4); 6 mg (*n* = 4); 8.4 mg (*n* = 9); 11.8 mg (*n* = 8); 16 mg (*n* = 9); and 22 mg (*n* = 7). Forty one patients were included in the DLT-evaluable population. No DLTs were observed in patients who received TAK-733 in the first eight dose cohorts (0.2–8.4 mg).

Subsequently, 4 patients experienced DLTs in cycle 1. In the 11.8 mg cohort, one of six DLT-evaluable patients had a DLT of grade 3 dermatitis acneiform on day 21 of cycle 1. The patient received oral minocycline, topical Neosporin, and topical clindamycin, and TAK-733 was discontinued; the event resolved approximately 1 week later. In the 16 mg cohort, one of seven DLT-evaluable patients had a DLT of grade 3 dermatitis acneiform on day 21. The patient received oral minocycline, topical clindamycin, and topical hydrocortisone, and TAK-733 was discontinued; the event was reported as ongoing at last follow-up. In the 22 mg cohort, one patient had grade 3 fatigue, pustular rash, and dermatitis acneiform on day 21 of cycle 1; TAK-733 was discontinued and the events resolved in approximately 2 weeks. A second patient had grade 2 dermatitis acneiform and stomatitis on day 12 of cycle 1; the TAK-733 dose was reduced to 16 mg and the events resolved in 3 and 8 weeks, respectively.

Based on the observed DLTs in cycle 1, the MTD of TAK-733 was determined to be 16 mg once daily on days 1–21 in 28-day treatment cycles.

### TAK-733 exposure and safety profile

Patients received a median of 2 cycles (range 1–11) of TAK-733; 7 (14 %) patients received ≥6 cycles. The mean total dose of TAK-733 received was 581.66 mg (ranging from 4.2 mg in the 0.2 mg dose level to 1425.71 mg in the 22.0 mg dose level), with a mean dose per week of 57.71 mg (ranging from 1.34 to 112.64 mg).

All patients experienced at least one AE of any grade, and 88 % reported drug-related AEs (Table 2). Grade ≥ 3 AEs were reported in 27 (53 %) patients, with drug-related grade ≥ 3 AEs reported in 12 (24 %). AEs of any grade reported in at least 20 % of patients, drug-related AEs reported in at least 10 % of patients, and all drug-related grade ≥ 3 AEs, are listed in Table 2.

**Table 2:** Safety profile of TAK-733, including AEs of any grade reported in at least 20 % of patients, drug-related AEs reported in at least 10 % of patients, and all drug-related grade ≥ 3 AEs. AE, adverse event; AST, aspartate aminotransferase; CPK, creatine phosphokinase

AE, *n* (%)	*N* = 51
Any AE	51 (100)
Common AEs (any grade; ≥20 % of patients)	
Dermatitis acneiform	28 (55)
Diarrhea	19 (37)
Fatigue	18 (35)
Peripheral edema	14 (27)
Increased AST	13 (25)
Increased CPK	10 (20)
Decreased appetite	10 (20)
Any drug-related AE	45 (88)
Common drug-related AE (≥10 % of patients)	
Dermatitis acneiform	26 (51)
Diarrhea	15 (29)
Increased blood CPK	10 (20)
Fatigue	9 (18)
Stomatitis	9 (18)
Peripheral edema	8 (16)
AST increased	7 (14)
Dry skin	5 (10)
Any grade ≥ 3 AE	27 (53)
Any drug-related grade ≥ 3 AE	12 (24)
Drug-related grade ≥ 3 AEs	
Increased blood CPK	5 (10)
Dermatitis acneiform	4 (8)
Stomatitis	1 (2)
Fatigue	1 (2)
Rash pustular	1 (2)
Hypophosphatemia	1 (2)
Pain in extremity	1 (2)
Neuropathy peripheral	1 (2)
Pulmonary embolism	1 (2)
Any serious AE	14 (27)
Any drug-related serious AE	1 (2)
AE resulting in study drug discontinuation	7 (14)
On-study death	5 (10)

**Fig. 1:**
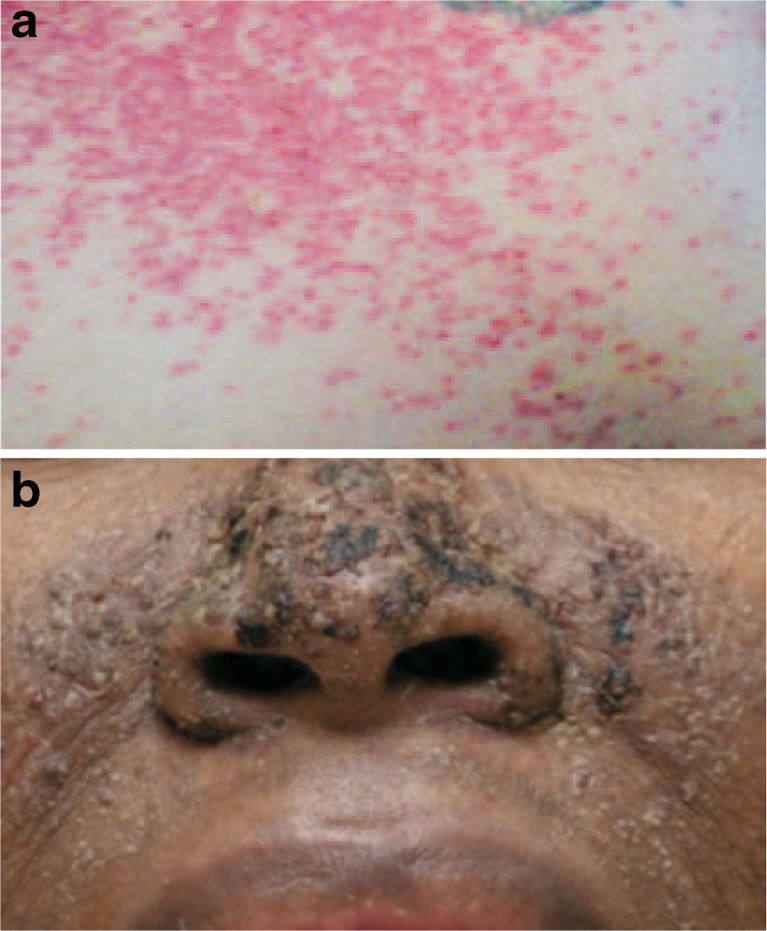
Dose-limiting toxicities (DLTs) of rash seen in patients receiving TAK-733. **a** Grade 3 dermatitis acneiform on day 21 of cycle 1 in a patient receiving TAK-733 11.8 mg – painful rash seen on scalp, face, and chest (shown in photo). **b** Grade 2 rash in a patient receiving TAK-733 22 mg that required dose reduction in cycle 1

Overall, 34 (67 %) patients experienced any rash AEs. This included 28 (55 %) with dermatitis acneiform, 3 (6 %) with rash, 2 (4 %) each with macular rash, maculo-papular rash, and papular rash, and 1 (2 %) each with exfoliative rash, erythematous rash, pruritic rash, and pustular rash. Two of the rash DLTs are shown in Fig. 1. The number of patients who experienced rash AEs increased with increasing dose of TAK-733; however, an analysis was conducted indicating no relationship between TAK-733 exposure and skin AEs (data not shown). No cutaneous squamous cell carcinomas were seen.

A total of 7 (14 %) patients experienced ophthalmic AEs within the eye disorders system organ class, including 1 at 8.4 mg, 2 at 16 mg, and 4 at 22 mg. These included visual impairment (*n* = 3), photopsia (*n* = 2), blurred vision (*n* = 2), abnormal sensation in eye, photophobia, periorbital edema, and retinal edema (each *n* = 1).

A total of 14 (27 %) patients experienced at least one serious AE (SAE), including 1 (2 %) who experienced drug-related SAEs. The only SAEs reported in more than one patient were progression of metastatic melanoma (*n* = 3), pulmonary embolism (*n* = 2), and anemia (*n* = 2). One patient experienced SAEs of hydroureter, hydronephrosis, and pulmonary embolism that were assessed as possibly related to TAK-733; the patient had a history of hydroureter and hydronephrosis at study entry.

Seven (14 %) patients experienced AEs that required TAK-733 discontinuation; these included: stomatitis in 1 patient at 8.4 mg; dermatitis acneiform in 1 patient at 11.8 mg and 1 patient at 16 mg; pneumonia in 1 patient at 16 mg; seborrheic dermatitis, swelling face, and fatigue in 1 patient at 16 mg; fatigue, pustular rash, and dermatitis acneiform (DLT) in 1 patient at 22 mg; and bacteremia in 1 patient at 22 mg. All AEs were considered related to TAK-733 except for pneumonia and bacteremia.

Five patients died on-study. None of the deaths were considered related to TAK-733, and all were due to the progression of the patients’ respective malignancies.

### Pharmacokinetics

**Fig. 2:**
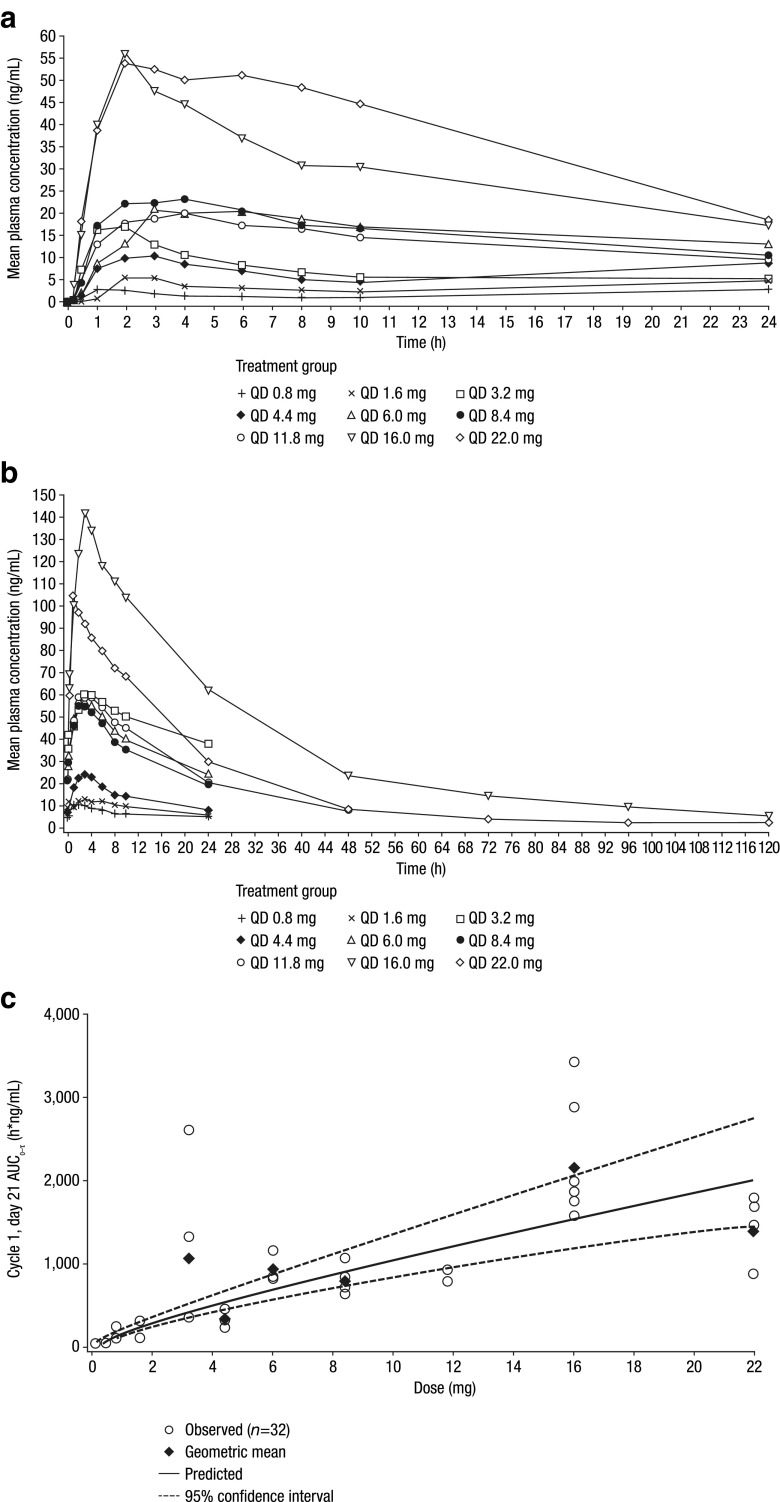
Pharmacokinetics of TAK-733 in the pharmacokinetic-evaluable population. Mean TAK-733 plasma concentration–time profiles on **a** day 1 and **b** day 21 of cycle 1, and **c** TAK-733 steady-state systemic exposure (day 21 AUC_0–τ_) versus dose (slope 0.8, 95 % CI: 0.63–0.98) indicating a less than dose-proportional relationship

TAK-733 plasma concentration–time data were available from all 51 patients who received TAK-733 over the dose range 0.2–22 mg; plasma concentration–time profiles on day 1 and day 21 of cycle 1 are shown in Fig. 2a and b. Following oral administration, absorption was fast, with an overall median T_max_ of 3 h (range 0.5–8.1) (Fig. 2a, b, Supplementary Table S1). Terminal half-life following multiple dosing ranged from 29 to 50 h over the 11.8–22 mg dose range, peak/trough ratio ranged from 1.3 to 4.8 (0.2–22 mg dose range), and the accumulation ratio varied from 0.9 to 9.0 across the 0.2–22 mg dose range. Dose-proportionality analysis of TAK-733 steady-state systemic exposure (AUC_0–τ_) versus dose showed a point estimate of the slope of 0.8 (95 % CI: 0.63, 0.98), indicating less than a dose-proportional increase over the TAK-733 dose range of 0.2–22 mg (Fig. 2c).

Urine pharmacokinetic data were available from 16 patients for estimation of the renal clearance of TAK-733. Renal clearance ranged from 0.05 to 0.49 L/h, which was 0.5 % to 4.8 % of the apparent oral clearance over the 0.2–22 mg dose range.

### Pharmacodynamics

**Fig. 3:**
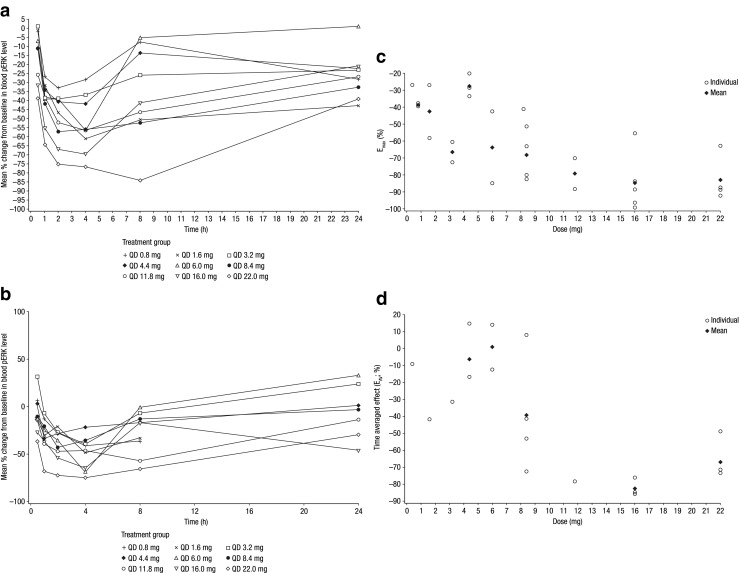
Pharmacodynamics of TAK-733 in the pharmacodynamic-evaluable population. Mean percent change from baseline in blood pERK levels versus time profiles (0–24 h) on **a** day 1 and **b** day 21 of cycle 1, and relationship between dose and (**c**) maximal pERK decrease (E_max_) and (**d**) steady-state time-averaged effect (E_av_) on day 21 of cycle 1

Data on the pharmacodynamic effects of TAK-733 (inhibition of ERK phosphorylation in peripheral blood mononuclear cells, as a biomarker of MEK inhibition) were available for 46 patients. Mean percent changes from baseline in blood pERK levels following single and multiple TAK-733 doses are shown in Fig. 3a, b. TE_max_ ranged from 0.8 to 24 h, with maximum decrease in pERK at day 21 of 46–97 % (E_max_) in patients receiving doses of 8.4 mg and above. On day 21 of cycle 1, the duration of pERK decreases appeared relatively transient at TAK-733 doses of less than 8.4 mg, while at higher doses target inhibition appeared to be sustained beyond 4 h (Fig. 3a, b). At the MTD of 16 mg, mean E_max_ was 85 %, and mean AUEC_(0-τ)_ was 1947 h* % on day 21 of cycle 1, translating to a mean E_av_ of 82 % (Table 3). Individual and mean values of E_max_ and time-averaged effect by TAK-733 dose at steady state (day 21) are shown in Fig. 3c, d, respectively. No analyses of correlations between pERK decreases and antitumor effects and no pharmacodynamic analyses at tumor sites were conducted as part of this study.

**Table 3:** Key TAK-733 pharmacodynamic parameters of decreases in blood pERK levels following oral administration of TAK-733 at doses of 0.8 to 22 mg in the pharmacodynamic-evaluable population. AUEC_(0-τ),_ area under the effect (inhibition of ERK phosphorylation)–time curve over the dosing interval; E_av_, average effect (inhibition of ERK phosphorylation) over the dosing interval; E_max_, maximum observed effect of inhibition of ERK phosphorylation; NR, not reported as n < 2; SD, standard deviation; TE_max_, time to E_max_

Dose, mg	*N*	Day	E_max_, %	TE_max_, h	AUEC_(0-τ)_, h*%	E_av_, %
			Mean (SD)	Median (range)	Mean (SD)	Mean (SD)
0.8	2	1	–40.55 (2.5)	3.0 (2–4)	–450 (60)	–19.3 (2.0)
		21	–39.2 (1.1)	3.0 (2–4)	NR	NR
1.6	2	1	–61.3 (7.0)	4.0 (4–4)	–1175 (134)	–47 (8.2)
		21	–43.0 (21.9)	NR	–1020	–42.2
3.2	3	1	–41.2 (7.2)	1.1 (1–4)	–643 (405)	–26.9 (16.9)
		21	–66.8 (8.4)	NR	–785	–31.6
4.4	4	1	–49.2 (3.7)	1.5 (1–2)	–542 (492)	–22.5 (20.5)
		21	–28.0 (6.8)	2.0 (2–4)	–164 (447)	–6.4 (18.3)
6	3	1	–59.8 (32.7)	4.0 (1–4)	–397 (458)	–16.5 (18.9)
		21	–64.0 (29.6)	NR	29 (464)	0.7 (18.7)
8.4	8	1	–64.5 (14.5)	3.0 (1–8)	–981 (624)	–41.6 (26.5)
		21	–63.8 (17.6)	2.1 (1–4)	–937 (811)	–39.7 (34.2)
11.8	7	1	–61.3 (11.4)	4.1 (4–8)	–971 (241)	–40.5 (10.0)
		21	–79.3 (12.7)	2.2 (2.1–2.3)	–1900	–78.1
16	9	1	–77.3 (6.7)	4.0 (2–8)	–948 (624)	–40.1 (26.3)
		21	–84.7 (17.3)	4.6 (4–24)	–1947 (125)	–82.4 (5.5)
22	7	1	–84.1 (9.0)	7.6 (1–8)	–1517 (257)	–64.5 (11.3)
		21	–82.9 (13.4)	3.1 (1–8)	–1615 (288)	–66.7 (11.7)

For AUEC_(0-τ)_, E_av_, and E_max_, the parameters are based on % change from baseline in pERK levels versus time, with inhibition of ERK phosphorylation being greater the more negative the number

### Antitumor activity

Among 41 response-evaluable patients, 2 (5 %) patients had partial responses. One patient (male, age 68 years) with BRAF L597R cutaneous melanoma, who received TAK-733 at 16 mg, had a partial response that was reported at cycle 4 and maintained until cycle 8 (approximate duration of 4 months). The patient had received three prior lines of therapy with interferon, granulocyte macrophage colony-stimulating factor (both as adjuvant therapy), and dacarbazine, as well as prior radiation therapy. A second patient (male, age 68 years) with cutaneous melanoma (mutation status not feasible to determine), who received TAK-733 at 22 mg, had a partial response at cycle 2 that was maintained until cycle 6 (approximate duration of 4 months). The patient had received one prior line of therapy with ipilimumab, to which his best response was progressive disease.

A further 15 (37 %) patients had a best response of stable disease, and the other 24 (59 %) evaluable patients had progressive disease. Two patients with melanoma who received TAK-733 at the 8.4 mg and 22 mg dose levels experienced stable disease lasting for 6 months or longer.

With a very small sample size, and low rate of responders, no correlation of mutation profile to response was seen in an analysis of archival tumor biopsies (data not shown).

## Discussion

This was the first-in-human study of TAK-733 in patients with advanced solid tumors. Our findings showed that the safety profile of TAK-733 was generally acceptable, with a manageable toxicity profile up to a dose of 16 mg QD for 21 days in 28-day cycles, which was determined to be the MTD (*n* = 9). Results from pharmacokinetic analyses showed that less than dose-proportional increases in steady-state exposures of TAK-733 were observed over the dose range of 0.2–22 mg, and, therefore, variability in dose-normalized exposures could not be assessed. A key finding of importance from the study was that sustained decreases in blood pERK levels were seen at higher doses of TAK-733; this anticipated pharmacodynamic effect of TAK-733 supports the mechanism of action of MEK1/2 inhibition. Also of note, we showed that two patients with cutaneous melanoma, including one with a BRAF L597R mutation, achieved partial responses that lasted approximately 4 months, suggesting evidence of preliminary antitumor activity with TAK-733.

TAK-733 represents one of several MEK1/2 inhibitors being investigated for the treatment of various cancers [16, 34]. Like the approved agent trametinib, as well as selumetinib [35] and other investigational MEK1/2 inhibitors, TAK-733 is a non-ATP competitive allosteric inhibitor of the MEK1 and MEK2 BRAF substrates [24]. The findings from the present phase I study appear generally consistent with data from early-phase clinical trials of these other MEK1/2 inhibitors, which, with the exception of trametinib, have generally demonstrated limited single-agent activity across multiple tumor types, along with similar DLTs and other toxicities [16, 34]. For example, the DLTs and/or most common drug-related AEs with TAK-733 in the present phase I study included dermatitis acneiform, diarrhea, increased blood creatine phosphokinase (CPK), fatigue, and stomatitis. These are similar to the common toxicities reported with other MEK1/2 inhibitors including trametinib [36–38], selumetinib [19], RO4987655 [39], and PD-0325901 [40]; trametinib has been reported to be specifically associated with acneiform eruptions [41]. Similarly, DLTs in other phase I studies of MEK1/2 inhibitors have included rash, diarrhea, increased blood CPK, and ocular toxicities with ARRY-424704, refametinib, cobimetinib, ARRY-438162, and pimasertib [16, 34]. These findings reflect the general association of dermatologic toxicities with small-molecule targeted cancer therapies such as kinase inhibitors, including MEK inhibitors [42, 43].

A notable aspect of the safety profile of TAK-733 was the limited rate of ophthalmic AEs, and the absence of retinopathies in the present study. Central serous-like retinopathy and serous retinal detachment, retinal vein occlusion, blurred vision, transient visual disturbance, and retinal pigment epithelial detachment have all been reported as DLTs with other MEK1/2 inhibitors in phase I clinical trials [16], with transient drug-related retinopathies reported with MEK inhibitors in patients with metastatic melanoma [37, 44] and MEK inhibitor treatment associated with other ocular adverse events [45]. These ocular toxic effects appear unique to MEK1/2 inhibitors [16], but did not appear to form a notable component of the TAK-733 safety profile.

For determination of plasma and urine pharmacokinetic profile, a validated LC-MS/MS method was employed and the TAK-733 concentrations were within the dynamic ranges of the assay (0.1–200 ng/mL for plasma and 5–10,000 ng/mL for urine). Findings from the analyses of TAK-733 pharmacokinetics in the present study indicated that exposures were attained that were in excess of those associated with antitumor activity in preclinical studies. For example, at the 16 mg dose of TAK-733, the geometric mean steady-state exposure (day 21 AUC_0–τ_) of 2154.4 h*ng/mL was approximately two-fold higher than the exposures associated with stasis in the most sensitive xenograft model treated with single-agent TAK-733 (861–1065 h*ng/mL) [46]. Furthermore, pharmacodynamic findings also showed that the mean E_av_ for inhibition of blood ERK phosphorylation at 16 mg (82 %) exceeded the lower bound of time-averaged inhibition of ERK phosphorylation (76–89 %) associated with tumor stasis in xenograft models treated with single-agent TAK-733 [46]. As was planned in the cancelled expansion stage, a more informative measure of the pharmacodynamic effects of TAK-733 would have been to measure its impact on pERK and downstream outcome markers (e.g. markers of proliferation and apoptosis) in matched tumor biopsies. Similar pharmacodynamic effects of target pathway inhibition, i.e. a decrease in pERK levels in peripheral blood mononuclear cells, malignant cells, and/or paired tumor biopsies, have been reported with other MEK1/2 inhibitors, including trametinib [37], selumetinib (79 % geometric mean pERK reduction in paired tumor biopsies [19, 22]), ARRY-424704 [47], and RO4987655 [39], and with the dual Raf/MEK inhibitor RO5126766 [48], although no correlations with response have been reported.

As with the safety profile and pharmacodynamic effects, the antitumor activity reported with TAK-733 in the present study appears consistent with that seen with most other MEK1/2 inhibitors in early-phase development [16, 34]. The objective response rate was limited, at 5 %, with responses including two partial responses in patients with cutaneous melanoma; additionally, two patients with melanoma achieved stable disease lasting for 6 months or longer. Notably, the response in a patient with BRAF L597R mutant melanoma is consistent with observations with other MEK1/2 inhibitors, including trametinib [36–38], which have shown activity in BRAF-mutant melanoma. Specifically, trametinib has been approved by the US FDA for the treatment of BRAF V600E/K-mutant melanoma, having demonstrated substantial response rates of approximately 20 % [36, 38] and improved progression-free survival and overall survival compared to chemotherapy with dacarbazine or paclitaxel [36] in this patient population. Subsequently, the combination of MEK1/2 inhibition with trametinib and the BRAF inhibitor dabrafenib has been FDA-approved based on enhanced responses and progression-free survival compared to dabrafenib monotherapy and improved overall survival compared to vemurafenib monotherapy [49, 50], indicating the importance of dual inhibition of the Ras/Raf/MEK/ERK pathway for improving antitumor activity, ameliorating paradoxical activation of MAPK signaling and partially overcoming potential resistance to single-target inhibition, e.g. BRAF inhibition.

In conclusion, the findings of the present phase I study of TAK-733 have demonstrated a safety profile, pharmacodynamic effects, and antitumor activity consistent with other MEK1/2 inhibitors, with key toxicities including dermatitis acneiform, diarrhea, increased blood CPK, fatigue, and stomatitis, and limited single-agent antitumor activity, including a partial response in a patient with BRAF-mutant melanoma. Given the limited antitumor activity, and in the context of data with trametinib and the recent changes in the standards of care in this indication, there are no plans for the future development of TAK-733 in patients with melanoma, and there are currently no other ongoing clinical studies of TAK-733.

## Electronic supplementary material

Supplementary Table S1 (DOCX 13 kb)
