# Supplementary material for: A phase I dose-escalation study of TAK-733, an investigational oral MEK inhibitor, in patients with advanced solid tumors
Source: Invest New Drugs. 2016 Sep 21;35(1):47–58. doi: 10.1007/s10637-016-0391-2 (PMC5306265; doi:10.1007/s10637-016-0391-2)
Supplement: Supplementary file 1 — (DOCX 13 kb) [file 10637_2016_391_MOESM1_ESM.docx]

**Supplementary materials**

**Supplementary Table S1.** Key TAK-733 plasma pharmacokinetic parameters following oral administration of TAK-733 at doses of 3.2 to
22 mg in the pharmacokinetics-evaluable population. %CV, coefficient of variation, NA, not applicable, ND, not determined, NR, not reported (as n<3), SD, standard deviation

| **Dose, mg** | ***N*** | **Day** | **C_max_, ng/mL**  **Geometric mean (%CV)** | **T_max_, h Median (range)** | **AUC_(0-τ)_, h*ng/mL Geometric mean (%CV)** | **Terminal t_½_, h**  **Mean (SD)** | **R_ac_**  **Mean (SD)** | **Peak/trough ratio**  **Mean (SD)** | **CL_ss_/F, L/h**  **Geometric mean (%CV)** |
| --- | --- | --- | --- | --- | --- | --- | --- | --- | --- |
| 3.2 | 4 | 1 | 14.8 (70) | 1.55 (1.1, 2.0) | 128.6 (90) | NA | NA | NA | NA |
|  |  | 21 | 63.0 (66) | 3.0 (3.0, 6.0) | 1051.4 (80) | ND | 7.2 (1.5) | 2.1 (0.8) | 3.0 (102) |
| 4.4 | 4 | 1 | 11.0 (40) | 3.5 (1.0, 6.0) | 98.1 (27) | NA | NA | NA | NA |
|  |  | 21 | 24.8 (40) | 1.95 (1.9, 2.0) | 317.9 (31) | ND | NR | 3.6 (1.1) | 13.8 (29) |
| 6 | 4 | 1 | 26.2 (40) | 4.5 (3.0, 8.0) | 331.1 (65) | NA | NA | NA | NA |
|  |  | 21 | 59.7 (17) | 3.0 (2.1, 4.0) | 925.2 (20) | ND | 2.8 (1.2) | 2.9 (1.2) | 6.5 (18) |
| 8.4 | 9 | 1 | 28.4 (34) | 3.0 (1.0, 6.0) | 360.4 (36) | NA | NA | NA | NA |
|  |  | 21 | 57.4 (11) | 3.0 (2.0, 4.2) | 785.6 (20) | ND | 2.5 (1.0) | 3.0 (0.8) | 10.7 (17) |
| 11.8 | 8 | 1 | 23.0 (46) | 3.05 (1.0, 8.0) | 298.9 (61) | NA | NA | NA | NA |
|  |  | 21 | 53.2 (7) | 4.0 (2.3, 4.3) | NR | 43.0 (7.3) | NR | NR | NR |
| 16 | 9 | 1 | 55.7 (43) | 2.1 (0.5, 4.4) | 640.3 (41) | NA | NA | NA | NA |
|  |  | 21 | 151.0 (24) | 3.4 (2.0, 6.0) | 2154.4 (33) | 39.9 (4.9) | 4.4 (2.6) | 2.9 (1.0) | 7.4 (28) |
| 22 | 7 | 1 | 60.0 (34) | 3.0 (1.0, 8.1) | 766.4 (42) | NA | NA | NA | NA |
|  |  | 21 | 104.6 (28) | 1.05 (1.0, 6.0) | 1394.5 (28) | 38.3 (6.9) | 1.8 (1.2) | 3.7 (0.6) | 15.7 (36) |
